# Quantification of circulating microRNAs by droplet digital PCR for cancer detection

**DOI:** 10.1186/s13104-020-05190-3

**Published:** 2020-07-23

**Authors:** Priscila D. R. Cirillo, Katia Margiotti, Alvaro Mesoraca, Claudio Giorlandino

**Affiliations:** 1 Human Genetics Laboratories, ALTAMEDICA, Altamedica Main Centre, Viale Liegi 45, 00198 Rome, Italy; 2 Department of Prenatal Diagnosis, ALTAMEDICA, Fetal-Maternal Medical Centre, Viale Liegi 45, 00198 Rome, Italy

**Keywords:** MicroRNA, Serum, Droplet digital PCR, Diagnostics, Cancer, Biomarker

## Abstract

**Objective:**

Circulating cell-free microRNAs (miRNAs) which consist of short-sequence RNAs are released from cells into the blood stream and has emerged as new biomarkers in the clinical cancer diagnosis and treatment. For instance, ovarian cancer comprises one of the three major malignant tumor types in the female reproductive system. The mortality rate of this cancer is the highest among all gynecological tumors, with ovarian cancer metastasis constituting an important cause of death. Therefore, development of a diagnostic tool that enables the ovarian cancer diagnosis in earlier stages is urgent.

**Results:**

We have described an efficient protocol for an accurate absolute quantification of circulating miRNAs in healthy and ovarian cancer serum samples. Our data showed that ddPCR methodology can accurately measure circulating miRNAs levels and that can be a useful tool in biomarkers discovery for ovarian cancer detection.

## Introduction

Early diagnosis is key to reducing cancer deaths. The discovery of small non-coding RNA sequences, around 17–27 nucleotides called microRNAs (miRNAs), has opened new opportunities for a non-invasive test for early cancer detection [1]. Accumulating evidences revealed that miRNAs are extensively involved in cancer progression and suppression by regulating thousands of cancer-associated genes [2]. Circulating cell-free miRNAs can stably exist not only in cytoplasm, but also in human biofluids. Previous studies has been shown that miRNAs levels has the potential to discriminate between healthy and diseased subjects in several cancer types [3–8]. In addition, differential miRNA expression has enabled the early diagnosis, predicted prognosis and response to therapy in cancer subjects [9]. These suggest that miRNA expression profiles clustering similar tumor profile more accurately than mRNA expression profile [10–12]. However, miRNA quantification in serum or plasma still lacks consistency and standardization and has been to challenging [13, 14]. In this study, we validated a method for the absolute quantification of circulating miRNAs, based on miRNA-specific LNA™ primers and DNA-binding dye detected by droplet digital PCR (ddPCR) technology recently commercially approved and with a literature support [15–17]. In particular, we validated this methodology to investigate (in the future) one specific panel of miRNAs as ovarian cancer biomarkers (multi-analyte combinatorial model). Ovarian cancer is the leading cause of gynecological cancer-associated deaths in developed countries. This high mortality rate of ovarian cancer is associated with the difficulties of early detection, because it is usually asymptomatic until late stage. If diagnosed at early stages (FIGO I-II), the 5-year survival rate exceeds 90%. In addition, an increasingly body of evidence have been shown that dysregulation of miRNAs is involved in ovarian cancer development and progression and that can be used as an important basis for diagnosis of ovarian cancer [18–20]. Here, we selected two exogenous (cel-miR-39-3p and UniSp6) and two endogenous miRNAs (miR-149-3p and miR-320a), for the methodological validation described above. We detected miR-320a as overexpressed miRNA in serum of ovarian cancer patients.

## Main text

### Material and methods

#### Serum samples

The local Ethics Committee (Artemisia S.p.A) has approved the protocol study. All health donors provided written informed consent for the use of their serum samples for research purposes. Serum samples from ovarian adenocarcinoma patients were purchased from several commercial biobanks (Discovery Life Science, BioIVT, and Victorian Cancer Bank). Five women without cancer (undergone a routine gynecological visit were selected as controls (C1-C5). Blood serum was collected from controls and also from 5 ovarian cancer patients (OV15, OV16, OV17, OV40, and OV41) before chemotherapy treatment and surgical resection. Three tumor patients were classified as high-grade serous carcinoma (OV16, OV17, and OV40) and 2 tumor patients (OV15 and OV41) as borderline ovarian serous cystadenoma.

#### RNA isolation

Five milliliters of blood were collected in Vacutest tubes (with cloat activator). Between 2–3 h after collection, tubes were centrifuged at 3000 rpm/4 °C/10 min. Serum (1 mL) were collected to a new 2 mL tube and re-centrifuged at 16000 g/4 °C/10 min. The supernatant was kept at − 80 °C until RNA isolation. RNA (including miRNA) was extracted from 200 μL of serum using the miRNeasy Serum/Plasma Advanced Kit (cat. no. 217204, QIAGEN) according to the manufacturer’s protocol with some variation regarding the exogenous RNA sequence as described below. After samples were mixed with buffer RPL, 3 µL (and not 3.5 μL) of a 0.2 nM miRNA cel-miR-39-3p (*C.elegans* sequence: 5′phosphorylated-UCACCGGGUGUAAAUCAGCUUG-3′) (Integrated DNA Technologies) was added. RNA samples (20 µL) were kept at −80 °C until cDNA synthesis by reverse transcription reaction.

#### Reverse transcription and ddPCR reaction

Measurements of circulating miRNA is not a feasible step [14, 21]. Thus, we fixed the volume of RNA template and serum volume. Four microliters of RNA collected from 200 µL of serum were reverse-transcribed according to the *microRNA PCR profiling using miRCURY LNA™ PCR primer sets* with the *QX200™ Droplet Digital™ PCR System protocol* recently released (QIAGEN). The circulating miR-320a has been already detected in breast and ovarian cancer patients even in early stages [17, 22] and was chosen to the technical validation step. The miR-149-3p it was chosen as putative endogenous normalizator circulating miRNA [22]. Minor changes were done as such as adding 0.2 µL of synthetic RNA spike-in UniSp6 during cDNA synthesis. For ddPCR reaction, the total cDNA was diluted 1:50 (for endogenous miRNAs detection) and 1:500 (for exogenous miRNAs detection). Briefly, 20 µL of PCR reaction containing 1µL of miRCURY LNA primers (QIAGEN) (singleplex, follow the catalogue numbers for cel-miR-39-3p [YP00203952], UniSp6 [YP00203954], miR-149-3p [YP00204093], and miR-320a [YP00206042]), 9 µL of diluted cDNA and 10 µL of 2 × EvaGreen Supermix (BioRad) were loaded into the droplet generator cartridge (BioRad). Then, 70 µL of droplet generation oil for EvaGreen (BioRad) was added at specific wells in the cartridge. The cartridge was transferred to the QX200 droplet generator (BioRad). After droplet generation, the droplets (40 µL) were loaded at 96-well PCR plate (BioRad) and the plate was heat-sealed with specific aluminum foil (BioRad). Thermal cycling conditions were as follow: 95 °C for 5 min, then 40 cycles of 95 °C for 30 s and 58 °C for 1 min (ramping rate reduced to 1.6 °C/s), and three final steps at 4 °C for 5 min, 90 °C for 5 min, and a 4 °C indefinite hold to enhance dye stabilization.

### Statistical analysis

All the statistical analysis were done using GraphPad Prism version 8.0.1. Pearson correlation test was applied to compare two independent datasets. Mann–Whitney test was used to compare the levels of miRNAs among cases and controls. A p-value less than 0.05 (*P* ≤ 0.05) was considered as statistically significant.

## Results and discussion

**Fig. 1:**
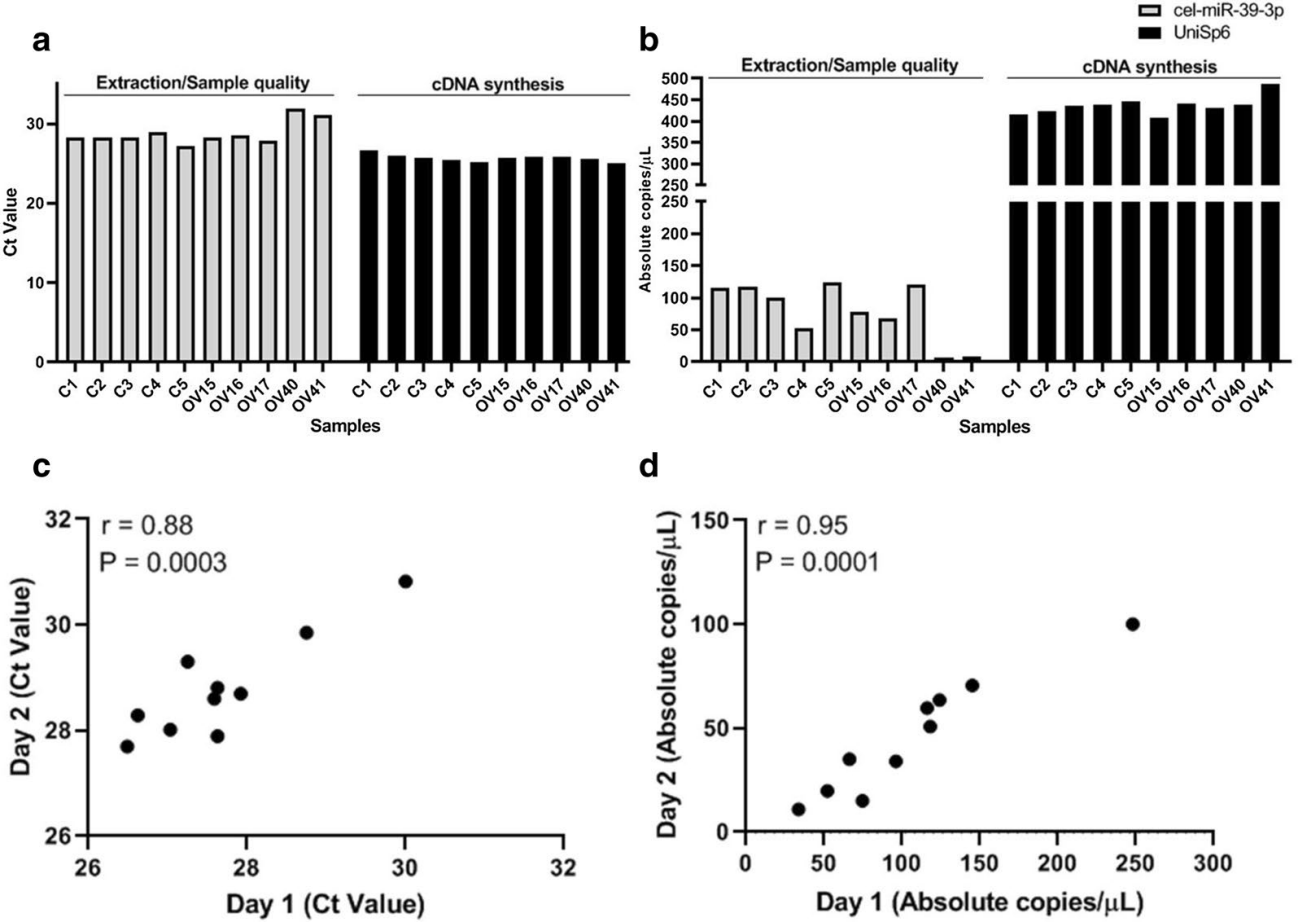
Optimization of cel-miR-39-3p and UniSp6 spike-ins and assay reproducibility for circulating miR-320a detection. All the 10 serum samples (5 from health-donors and 5 from ovarian cancer patients) were spiked-in with 0.2 nM of cel-miR-39-3p (gray bars) during extraction step and 0.2 µL of UniSp6 (black bars) were add during the cDNA synthesis. **a** RT-qPCR and **b** ddPCR were used to quantify the levels of both miRNAs. For the circulating miR-320a quantification, Pearson correlation (r) was applied to 2 independent datasets for all 10 samples to calculate the reproducibility of detection of miR-320a by (**c**) RT-qPCR and (**d**) ddPCR. “Day 1” and “Day 2” means two independent experiment (2 independent batches). *Ct* cycle threshold, *C* Health-controls, *OV* ovarian cancer patients

In our study, we were able to validate a recently approved commercial protocol for absolute quantification of circulating miRNAs (two endogenous and two exogenous miRNAs) in serum obtained from healthy donors and subjects affected by ovarian cancer. Quantification of cell-free miRNAs has recently come to the forefront as a potential source of cancer disease biomarkers. Nevertheless, miRNA quantification has been detected in a variety of manners, thus generating poorly reproducible results [23]. Standardization of methods for validation of biomarkers from basic research to clinical research is the key. The protocol based on high-specific miRNA primers and DNA-binding dye detection by ddPCR, has also been applied by others studies, with a high intra and inter-assay reproducibility [15–17]. Here, we used the ddPCR technology to get an accurate absolute quantification of specific circulating miRNAs. Firstly, the concentration of cel-miR-39-3p was defined by doing a serial dilution (seven points: 1:10) of spiked-in serum (two different samples) with an initial amount of 4.16 nM. This exogenous synthetic miRNA has been used for monitoring the efficiency of RNA extraction and sample quality [24]. The limit of detection (LOD) of cel-miR-39-3p was 1.12 ± 0.16 copies/µL and the optimal concentration of 0.2 nM (cDNA diluted 1:500) was found without saturation of positive droplets (data not shown). After that, all the 10 serum samples were spiked-in with 0.2 nM of cel-miR-39-3p during the extraction step. qRT-PCR analysis revealed that the levels of cel-miR-39-3p for most of the samples were around cycle threshold (Ct) value of 28 (Fig. 1a), except for samples OV40 and OV41 (Ct 31). Interestingly, using the same cDNA preparation, the quantification of cel-miR-39-3p by ddPCR were more similar between the most of the samples (around 100 copies/µL) but more variable among the samples OV40 and OV41, indicating that these samples could had some pre-analytical issue, leading to a decrease of the concentration of the cel-miR-39-3p (Fig. 1b), consistently with the RT-qPCR late Ct. Particularly, these both samples showed clear signals of hemolysis, which can also influence the detection of circulating miRNAs [24]. Pre-analytical condition is a crucial step for an accurate quantification of circulating miRNA. Another synthetic miRNA, the UniSp6, was used to monitor the efficiency of cDNA synthesis and the input of the template. The Fig. 1a, b shown that the levels of UniSp6 not floated, indicating the similar efficiency of cDNA synthesis and same input of template among the samples. Based on exogenous miRNAs results, we moved our analysis to the endogenous miRNAs data.

As cited before, the miR-149-3p and miR-320a were chosen for our validation based on previous results indicating them as putative endogenous normalizator and association with ovarian cancer early detection, respectively [22]. Our results revealed that the miR-149-3p were not detected in any samples by qRT-PCR (two independent assays), instead the ddPCR detected miR-149-3p levels down to LOD (1 copies/µL) (Additional file 1: Figure S1). These results confirm the high sensitivity of ddPCR to detect low target levels. However, this miRNA is not a good endogenous normalizator for our purpose, due to its downregulation. Thus, we decided to use the exogenous cel-miR-39-3p and UniSp6 as normalizators as suggested by Vigneron et al. [25]. For miR-320a, two independent assays were done in different batches (since from extraction step) for both RT-qPCR and ddPCR. The results showed that the assay reproducibility by RT-qPCR was r = 0.88 (Pearson correlation) (Fig. 1c), whereas the ddPCR assay reproducibility was r = 0.95 (Fig. 1d), indicating the higher performance of ddPCR in comparison to RT-qPCR. Together, these results indicate the applicability of ddPCR for our future purposes.

**Fig. 2:**
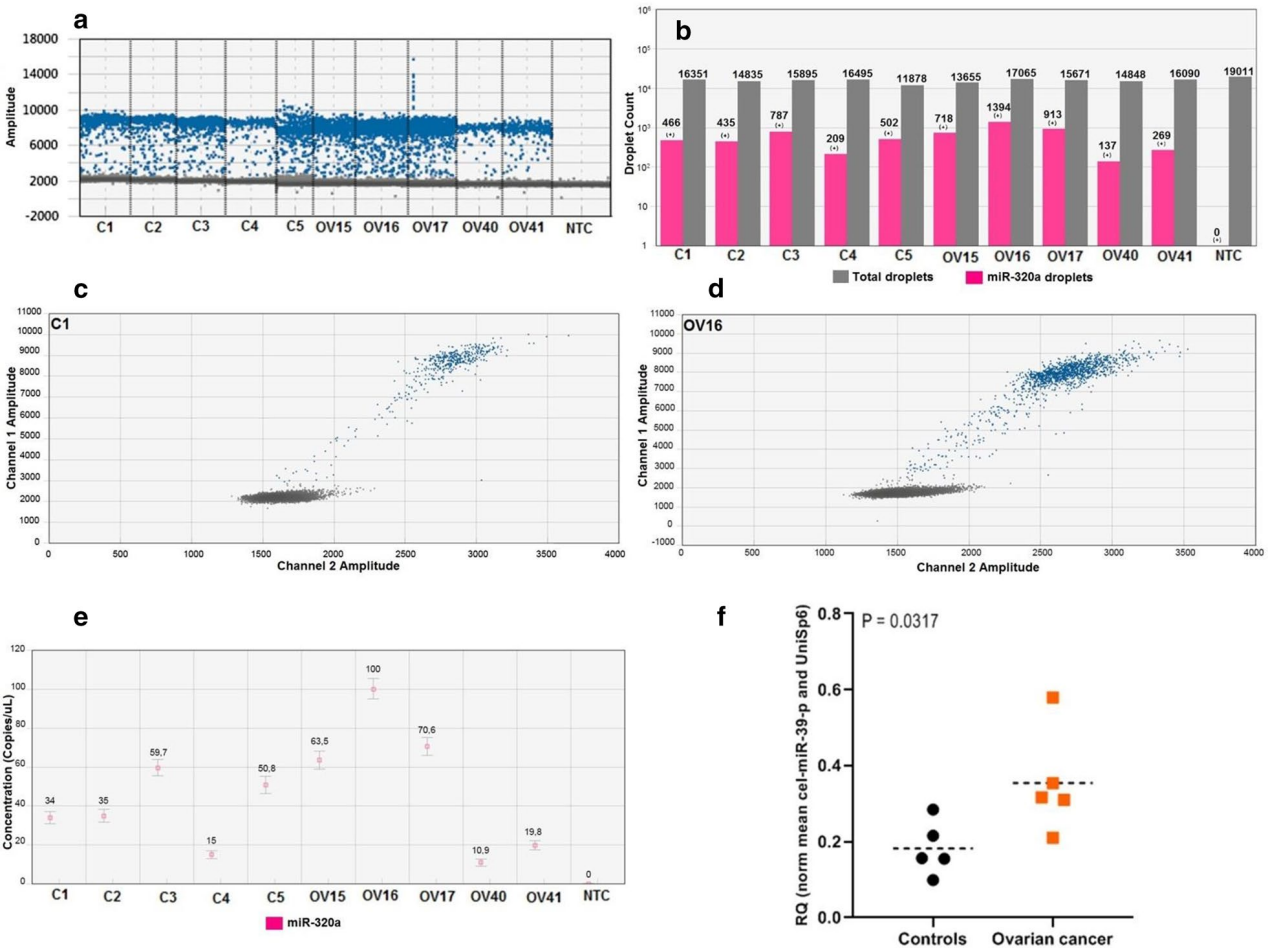
ddPCR quality control parameters for an accurate quantification of circulating miR-320a. All the 10 serum samples (5 from health-donors and 5 from ovarian cancer patients) were submitted to a ddPCR reaction whereas the quality control parameters were evaluated. **a** Representative 1D plot showing the effect of the annealing temperature at 58 °C and 1 µL of LNA primer volume on the amplitude of positive (blue) and negative (gray) droplets. No template control (NTC) has not positive droplets. **b** All samples had more than 10,000 total droplets detected even in a single-plex (gray bars), and a minimum  number of positive droplets ≥ 3 was considered to call a positive sample for miR-320a detection (pink bars). NTC (Negative Template Control) had 0 droplets indicating no false-positive droplets calling. **c**, **d** Bi-dimensional plots showing the “raining droplets” (blue droplets between 3000–8000 amplitude), a typical appearance of positive droplets detected for miRCURY LNA primers combined with EvaGreen-based assays. Around 10,000 amplitude is possible to observe one accumulation of miR-320a positive droplets in ovarian cancer case (**d**) (OV16) compared to the control (**c**) (C1). **e** Absolute quantification (concentration in copies/µL) of miR-320a for all 10 samples. **f** Mann–Whitney test was applied for all 10 samples for miR-320a quantification after normalization considering the geometric mean of cel-miR-39–39 and UniSp6. The miR-320a expression profile was able to distinguish health-controls from ovarian cancer serum samples (*P* = 0.037). *C* Health-controls, *OV* ovarian cancer patients, *RQ* Relative quantification. Error bars = Poisson 95% confidence interval. Representative data from 2 independent experiments

The primer optimization is one of the crucial steps for an accurate separation of negative from positive droplets in the ddPCR [16]. For all miRNAs validated here, the annealing temperature of 58 °C and 1µL of primer assay generated good separation of the droplets (Fig. 2a, c, d). However, for each new target, one step of primer optimization should be done in a small number of serum samples. In addition, for all samples, the number of total droplets reached ≥ 10,000 per well and a minimum of positive droplets ≥ 3 was considered to call a positive sample (Fig. 2b), which is the minimum acceptable value for the ddPCR Poisson precision calculation. All the assays were done with at least one NTC (No Template Control). The samples were considered positives after a confirmation of NTC results showing less than 3 positive droplets. This is a recommended practice to avoid the false-positive calls. Notable, it was clear to observe the high accumulation of positive droplets for miR-320a for the ovarian cancer samples OV15, OV15 and OV16, compared to the controls (Fig. 2a, c, d). After the Poisson calculation, the number of positive droplets were converted to copies/µL (absolute quantification) (Fig. 2e), where is possible to see the miR-320a expression profile between the samples. Finally, after normalization using mean of cel-miR-39-3p and UniSp6 levels and applying the Mann–Whitney test, we can observe that the miR-320a expression levels was able to differentiate ovarian cancer patients from healthy controls (*P* = 0.0317) (Fig. 2f), indicating the potential use of this miRNA among a specific miRNAs panel as diagnostic biomarkers for ovarian cancer detection.

## Conclusion

Overall, the present study reported the feasibility of using ddPCR for circulating miRNA absolute quantification and its potential use as a tool to help the clinicians to detect ovarian cancer in a group of high-risk patients.

## Limitations

Some limitations referred to the number of miRNAs utilized; more miRNAs should be tested in a high number of cases and controls to better define the clinical application of this novel technology.

## Supplementary information

**Additional file 1: Figure S1** Absolute ddPCR quantification of miR-149-3p.

## Data Availability

Not applicable.

## Abbreviations

miRNAs: microRNAs
ddPCR: droplet digital PCR
LOD: Limit of detection
