# Supplementary material for: Quantification of circulating microRNAs by droplet digital PCR for cancer detection
Source: BMC Res Notes. 2020 Jul 23;13:351. doi: 10.1186/s13104-020-05190-3 (PMC7379807; doi:10.1186/s13104-020-05190-3)
Supplement: Supplementary file 1 — Additional file 1: Figure S1 Absolute ddPCR quantification of miR-149-3p. [file 13104_2020_5190_MOESM1_ESM.pptx]

## Slide 1
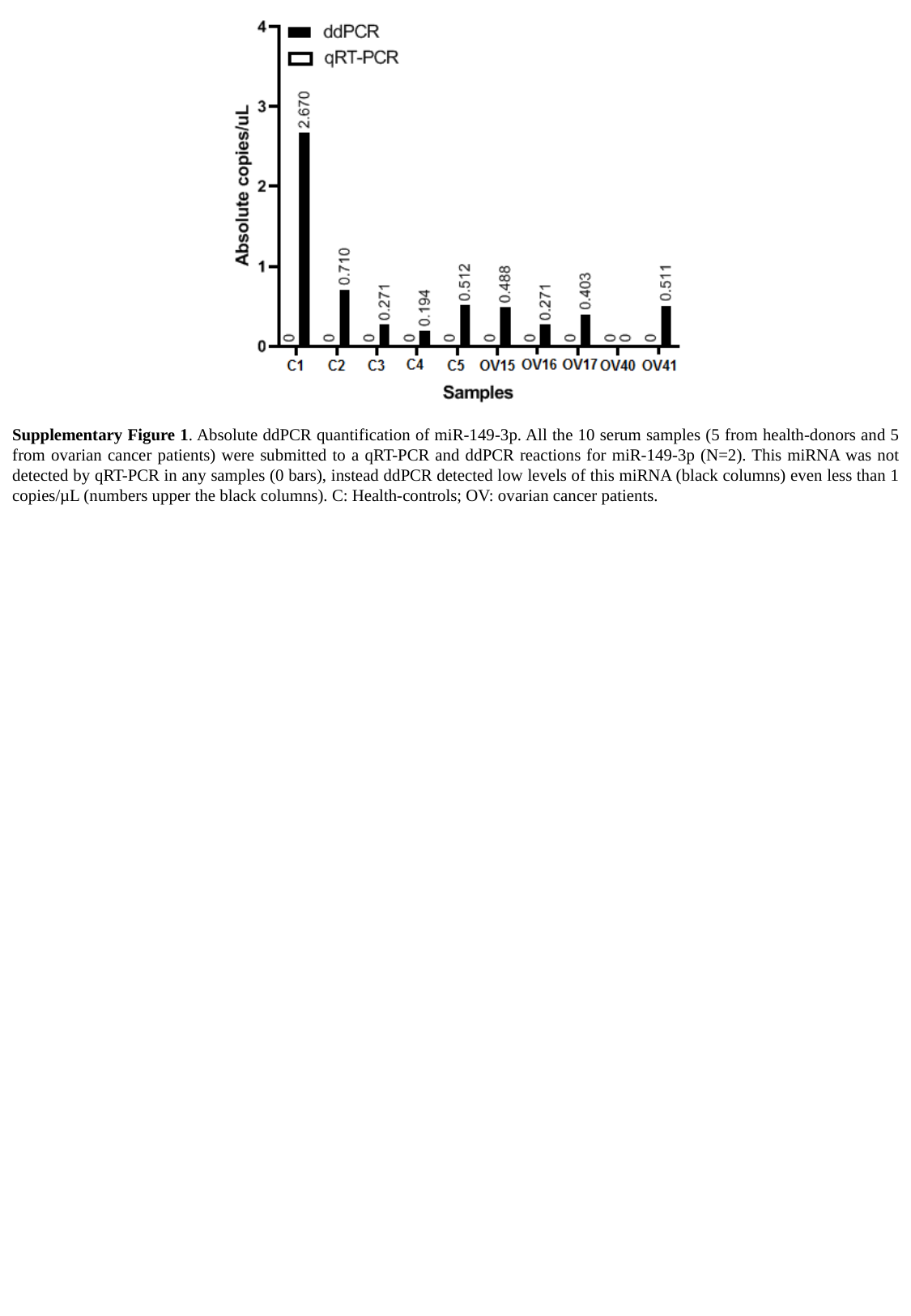

Supplementary Figure 1. Absolute ddPCR quantification of miR-149-3p. All the 10 serum samples (5 from health-donors and 5 from ovarian cancer patients) were submitted to a qRT-PCR and ddPCR reactions for miR-149-3p (N=2). This miRNA was not detected by qRT-PCR in any samples (0 bars), instead ddPCR detected low levels of this miRNA (black columns) even less than 1 copies/µL (numbers upper the black columns). C: Health-controls; OV: ovarian cancer patients.
